# Phenoxybenzamine in Complex Regional Pain Syndrome: Potential Role and Novel Mechanisms

**DOI:** 10.1155/2013/978615

**Published:** 2013-12-19

**Authors:** Mario A. Inchiosa

**Affiliations:** Departments of Pharmacology and Anesthesiology, New York Medical College, Basic Sciences Building, Valhalla, NY 10595, USA

## Abstract

There is a relatively long history of the use of the **α**-adrenergic antagonist, phenoxybenzamine, for the treatment of complex regional pain syndrome (CRPS). One form of this syndrome, CRPS I, was originally termed reflex sympathetic dystrophy (RSD) because of an apparent dysregulation of the sympathetic nervous system in the region of an extremity that had been subjected to an injury or surgical procedure. The syndrome develops in the absence of any apparent continuation of the inciting trauma. Hallmarks of the condition are allodynia (pain perceived from a nonpainful stimulus) and hyperalgesia (exaggerated pain response to a painful stimulus). In addition to severe, unremitting burning pain, the affected limb is typically warm and edematous in the early weeks after trauma but then progresses to a primarily cold, dry limb in later weeks and months. The later stages are frequently characterized by changes to skin texture and nail deformities, hypertrichosis, muscle atrophy, and bone demineralization. Earlier treatments of CRPS syndromes were primarily focused on blocking sympathetic outflow to an affected extremity. The use of an **α**-adrenergic antagonist such as phenoxybenzamine followed from this perspective. However, the current consensus on the etiology of CRPS favors an interpretation of the symptomatology as an evidence of decreased sympathetic activity to the injured limb and a resulting upregulation of adrenergic sensitivity. The clinical use of phenoxybenzamine for the treatment of CRPS is reviewed, and mechanisms of action that include potential immunomodulatory/anti-inflammatory effects are presented. Also, a recent study identified phenoxybenzamine as a potential intervention for pain mediation from its effects on gene expression in human cell lines; on this basis, it was tested and found to be capable of reducing pain behavior in a classical animal model of chronic pain.

## 1. Introduction

Complex regional pain syndrome (CRPS), types I and II, is the current accepted nomenclature for related neuropathic pain syndromes that typically develop following injury or surgery to an extremity [1]. Type I designation originally referred to conditions that were not believed to have been associated with nerve injury; this condition was previously called reflex sympathetic dystrophy (RSD). Type II CRPS was earlier termed causalgia and was associated with clear evidence of nerve damage [2]. In view of evidence that small nociceptive fibers may be damaged in CRPS I, the validity of an important distinction between type I and type II syndromes, in terms of etiology, symptomatology, and therapeutic approaches, is controversial [3–5]. No effort will be made to address this controversy, since it may not be relevant to the possible value of phenoxybenzamine for the treatment of CRPS.

Phenoxybenzamine is a noncompetitive (irreversible) antagonist of *α*_1_- and *α*_2_-adrenergic receptors [6]. It forms covalent bonds with these receptors, which results in a duration of blockade of 8 days or longer for full recovery of adrenergic effects on blood pressure (animal experiments) [7]. The irreversible action is the basis for its use in the control of hypertensive crises in patients with pheochromocytomas, tumors of the adrenal medulla that secrete massive amounts of catecholamines [8].

## 2. Current Therapy for CRPS

Although numerous drugs and interventions have been tried in attempts to treat CRPS, reliable relief of pain, restoration of function, and a “cure” remain difficult challenges. Two analyses have attempted to develop evidence-based guidelines for the treatment of CRPS. One covers trials in the period between 1980 and June 2005 [9]; the second covers the period from June 2000 to February 2012 [10]. The earlier review identified the following treatments that had varying degrees of positive therapeutic effect: subanesthetic ketamine intravenous infusion (10 to 50 mg/hr, usually over several days); gabapentin (600 to 1800 mg daily); dimethyl sulphoxide (50% cream applied to an affected area); N-acetylcysteine (600 mg three times daily); oral corticosteroids; bisphosphonates (e.g., alendronate 40 mg/day); nifedipine (20 mg/day); spinal cord stimulation (in selected patients); and various physiotherapy regimens.

Positive findings to varying degrees in the more recent analysis were as follows [10]: low-dose ketamine infusions (continuous for 4.5 days or as outpatient for 10 consecutive working days); bisphosphonates; oral tadalafil (20 mg per day, 12 weeks); intravenous regional block with a mixture of 5 mg parecoxib, 1 mg/kg lidocaine, and 30 *μ*g/kg clonidine; intravenous immunoglobulin (0.5 mg/kg); memantine (n-methyl-d-aspartate antagonist) 40 mg per day (with morphine); and physiotherapy. Spinal cord stimulation and transcranial magnetic stimulation improved symptoms, but only transiently.

Phenoxybenzamine has been studied in CRPS because of its potential to modify sympathetic nerve effects, perhaps, particularly, for its capacity to possibly improve blood perfusion in the cold limb of the chronic CRPS patient. The drug is not labeled by the United States Food and Drug Administration (FDA) for treatment of CRPS. However, in relation to its vasodilating effects, there is an off-label indication for phenoxybenzamine in the treatment of peripheral vascular diseases (e.g., Raynaud's disease) in the official American Hospital Formulary Service Compendium [11].

## 3. Clinical Experiences with Phenoxybenzamine in CRPS

Bennett and Brookoff [12] have traced the recognition of posttraumatic neuropathic pain syndromes to antiquity and have compiled the record of reports of such syndromes during the United States Civil War and the First and Second World Wars. It is of interest that probably the first detailed report of the clinical use of phenoxybenzamine for the treatment of CRPS (designated causalgia, type II, in this case) was from Walter Reed Army Hospital; all of the pain syndromes followed injuries, some of which occurred in armed conflict [13]. Eleven of 15 patients that were treated with phenoxybenzamine appeared to obtain temporary or permanent relief of their symptoms to varying extents. A more recent report on the use of phenoxybenzamine by Ghostine et al. [14] was also for treatment of causalgia associated with battlefield wounds to soldiers. The Ghostine study included a total of 40 consecutive patients over a period of 7 years who presented with neuropathic pain resulting from missile or shrapnel wounds. Dosage escalations were used to obtain pain relief and then continued for a period of usually 6 to 8 weeks. The oral daily dose ranged from 40 to 120 mg per day, which is a large dose in terms of expected occurrence of postural hypotension. This side effect occurred in 17 of the patients but resolved after approximately one week or was controlled with vascular support wrappings. In no case was it necessary to stop the treatment. The authors reported a complete resolution of the pain syndrome in all of the cases [14].

Muizelaar et al. [15] reported their retrospective experience for the treatment of CRPS (both type I and type II) with phenoxybenzamine over an 8-year period. Of a total of 33 patients that received phenoxybenzamine, 17 (52%) improved to the point where they were able to return to their former employment or resume social activities and were no longer using any narcotic medications. The success rate was higher (8 of 9; 89%) in the early and intermediate stages of the syndrome (2–7 months) than in the later chronic stages (9 of 24; 38%). Large doses were again used in this study. The dose was escalated, as tolerated, over a period of 8 to 11 days to a maximum daily oral dose of 120 mg, taken in 3 divided doses. The side effects included, to varying degrees, symptoms of orthostatic hypotension and general malaise in almost all of the cases, and impotence in males. Treatment was usually continued for 8 to 12 weeks. Seven of the 33 patients withdrew from treatment because of the side effects.

Phenoxybenzamine was used in the treatment of a case of CRPS (RSD) of the lower extremity in a patient with long-standing tetraplegia who sustained an apparent ankle injury during a transfer maneuver [16]. The lower limb became markedly edematous, warm, and painful. The diagnosis of CRPS was made on the basis of bone scintigraphic techniques that have high sensitivities and specificities for a neuropathic syndrome. Initial treatment with indomethacin was replaced with oral phenoxybenzamine at 10 mg per day for 4 days, followed by 10 mg twice a day. Both the edema and the pain in the lower extremity resolved within a few days of treatment. The phenoxybenzamine was continued for 3 months and was then tapered off with no recurrence of symptoms. Scintigraphic scans that were repeated after 30 months showed no differences between the previously involved limb and the contralateral limb.

In view of the presumed involvement of the sympathetic nervous system in CRPS, we initiated a clinical trial to use phenoxybenzamine as part of an intravenous regional block of an affected extremity [17]. Since there was no FDA-approved intravenous preparation of phenoxybenzamine or an approved use for CRPS, we conducted our study under an FDA-approved Investigational New Drug (IND) Application. We studied 5 patients with diagnoses of CRPS of an upper extremity that had not responded to conventional therapeutic approaches. The duration of their illnesses before our intervention had ranged from 2 to 60 months. All of the patients entered our study after failures with extensive drug treatments, mostly nonsteroidal anti-inflammatory drugs (NSAIDs) and opioids, and physical therapy. In addition, the patient with the longest disease history had received multiple stellate ganglion blocks, intravenous regional lidocaine blockade, trigger point injections, and cervical epidural steroids.

**Figure 1:**
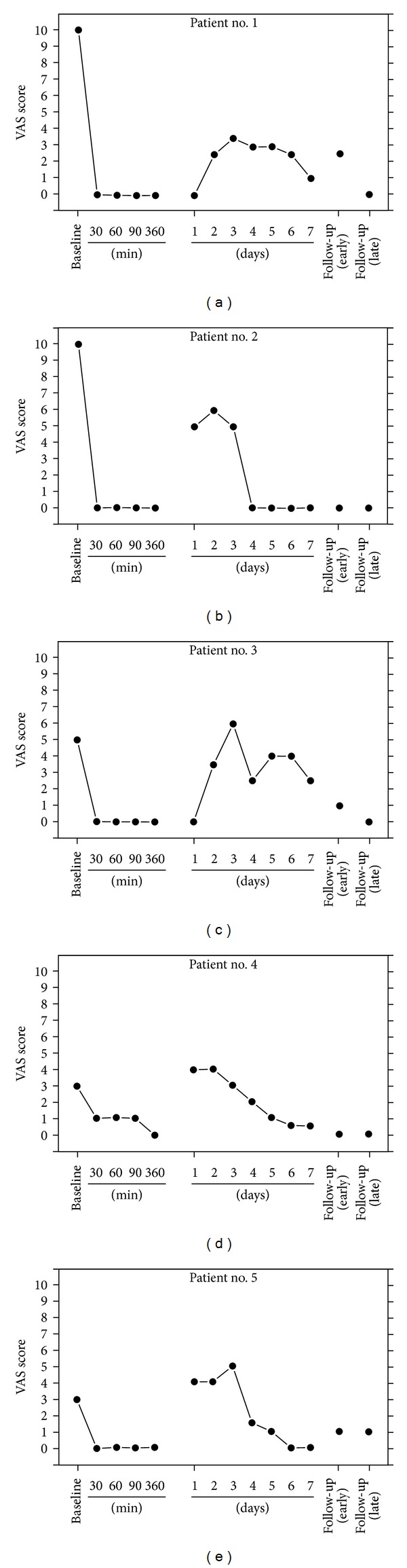
Baseline and postprocedure VAS scores for patients treated by intravenous regional blockade with phenoxybenzamine. Early follow-up evaluation times were at 6, 8, 11, 27, and 11 days after the procedure for patients 1, 2, 3, 4, and 5, respectively. The latest follow-up evaluations were at 17, 7, 5, 9, and 7 months for patients 1, 2, 3, 4, and 5, respectively. Reproduced with permission from *Anesthesiology*, 1998 [17].

Partial exsanguination of the limb was accomplished with the aid of a tourniquet proximal to the pathologically involved area, elevation of the limb, and application of an Esmarch bandage to facilitate venous drainage. The tourniquet pressure was set at 100 mmHg above systolic pressure of the extremity. The block solution consisted of 15 mL of 0.5% lidocaine HCl, 5 mg of phenoxybenzamine HCl (from sterile solution, 50 mg/mL; SmithKline-Beecham Pharmaceuticals, King of Prussia, PA), plus isotonic saline to a total volume of 30 mL. The solution was infiltrated over a period of 4 min; the tourniquet was deflated after a minimum period of 15 min. Each of the patients responded favorably to a single intravenous block containing phenoxybenzamine in the affected extremity. Visual analog pain (VAS) scores were considerably reduced one week after the block and remained close to complete reversal of pain at early (6 to 27 days) and late (5 to 17 months) follow-up evaluations (Figure 1). Skin temperature was increased by an average of 2.6 deg C in the treated arm over the pretreatment reading at the early follow-up evaluation but did not reach statistical significance. Hand-grip strength was significantly increased (*P* < 0.05) by an average of 9.9 kg over the pretreatment measurement at the early follow-up, as well. (Late follow-up measurements of skin temperature and hand-grip strength were not obtained; VAS scores were collected by telephone interview.) The experimental design might be criticized in that a local anesthetic was part of the block and that this could account for the apparent success that was seen. But a recent review on evidence-based treatment of CRPS I drew the strong conclusion that there is no evidence of therapeutic efficacy for intravenous sympathetic blockade [9]. However, it might still be allowed that there was some possible synergism elicited by the mixture of local anesthetic and phenoxybenzamine in our treatments. In fact, the need for a multimodal approach may prove to be optimal in the treatment of a syndrome that has such a complex mediation [18].

Soon after the report of our study with phenoxybenzamine as part of a regional sympathetic block, we lost complete availability of a source of phenoxybenzamine for intravenous administration. There is still no preparation of the drug at this time for intravenous injection in the United States. In an effort to be responsive to inquiries from patients with CRPS, we suggested oral treatment with phenoxybenzamine; this preparation is readily available. We have reported the experience with 4 patients that were treated with oral phenoxybenzamine and followed up for many months [19]. Since all the patients had long-standing disease before starting phenoxybenzamine treatment (13 and 20 months, and 5 and 6 years), they had received multiple drug treatments and interventions. These included oral and spinal opioids, antidepressants, multiple intravenous regional blocks with guanethidine, ankle blocks, and acupuncture. Two of the patients continued some medications in conjunction with phenoxybenzamine; more complete histories, treatments, and responses are detailed in the original report [19]. Three of the patients fulfilled classic International Association for the Study of Pain (IASP) criteria for diagnosis of CRPS type I [1, 20]. One patient did not demonstrate the classic syndrome, and we were not involved with the decision by the patient and her physician to test a course of treatment with the drug. This patient was the only one who did not have an apparent therapeutic response to the drug. The two patients who were the most severely incapacitated by disease at an advanced stage essentially returned to their predisease states. One patient had a partial, substantial improvement. She continues to take the drug at 10 mg every third day; at the time of this writing, it represents a treatment period of more than 12 years.

In comparison with the large doses of phenoxybenzamine that were used in studies noted above [14, 15], the apparent therapeutic benefit in our study was obtained with doses that did not exceed 10 mg/day, initially, and finally tapered to as low as 10 mg every third day for two of the patients. The one patient who did not have a diagnosis of CRPS and did not have a positive response eventually increased her dose to 30 mg/day before discontinuing treatment after 6 weeks. It is of interest that she did not report side effects during the treatment.

The advantage of maintenance with low doses was the avoidance of the side effects that may accompany treatment with an *α*-adrenergic antagonist, in particular, orthostatic hypotension. Some symptoms of light-headedness and nausea were recorded with the first initial doses of 10 mg/day, but tolerance soon developed to these effects.

The results from the several clinical trials for treatment of CRPS with phenoxybenzamine are summarized in Table 1.

## 4. Potential Mechanisms of Action in CRPS

We have attempted to suggest a rationale for the efficacy of phenoxybenzamine in the treatment of CRPS that includes its noncompetitive (irreversible) block of both *α*_1_- and *α*_2_-adrenergic receptors [6]. The long duration of blockade of receptor access may facilitate a reversal of central nervous system sensitization, which is an accepted component of the syndrome [2]. Even in the study where the drug was administered intravenously at a total dose of 5 mg [17], the drug would have continued to be effective in the limb where it was infused (and perhaps in a wider distribution after release of the tourniquet) for many days.

We have suggested a possible multimodal mechanism of action for phenoxybenzamine in CRPS. The drug may be capable of acutely diminishing the *α*_1_-adrenergic-mediated vasoconstriction that is seen in the later stages of the syndrome and the hyperalgesic state, both of which are generally attributed to receptor upregulation on the vasculature and afferent sensory fibers, respectively [21, 22]. It is generally considered that the supersensitivity in CRPS is a compensatory consequence of decreased sympathetic outflow to the traumatized extremity [2, 23, 24]. The early responses to phenoxybenzamine that we observed are consistent with relatively prompt evidence of improved vascular perfusion of the limb (increased skin temperature and reduction of edema) and diminution in pain [17, 25]. In addition to these early effects, we have suggested that phenoxybenzamine may exert an “immunomodulatory/anti-inflammatory” effect that may be part of its longer term contribution to the resolution of the neuropathic syndrome [19]. These effects may involve noncompetitive blockade of *α*_2_-adrenergic receptors on the surface membrane of immune elements, specifically, macrophages. The agonist activity of norepinephrine (and epinephrine) on *α*_2_-receptors of macrophages is reported to enhance the release of the inflammatory cytokine, tumor necrosis factor alpha (TNF-*α*) [26, 27]. TNF-*α*, substance P, and interleukin 1*β* are all inflammatory cytokines that sensitize afferent sensory neurons [2, 23, 28, 29]. In this connection, phenoxybenzamine has been shown to decrease the release of TNF-*α* from macrophages in endotoxin-challenged mice [30, 31].

**Figure 2:**
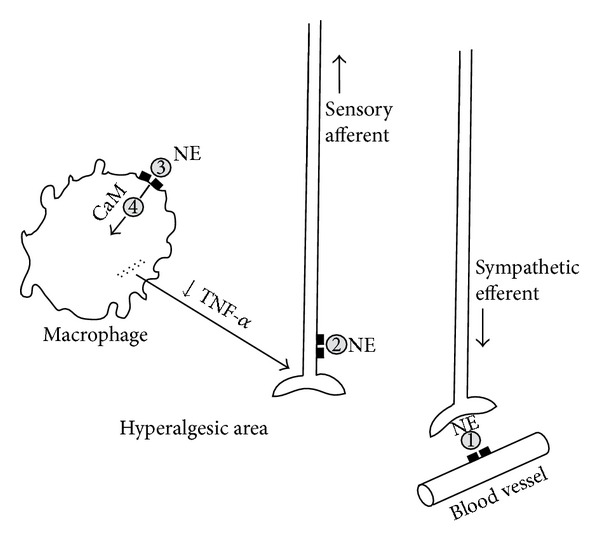
Depiction of possible sites of action of phenoxybenzamine in the suppression of neuropathic pain. Site 1) Blockade of norepinephrine (NE) effects on *α*_1_-adrenergic receptors on blood vessels, thereby promoting vasodilation; Site 2) blockade of adrenergic receptors that populate afferent sensory fibers; Site 3) blockade of *α*_2_-adrenergic receptors on the surface of macrophages, which appear to mediate release of proinflammatory cytokines, including tumor necrosis factor-*α* (TNF-*α*); Site 4) inhibition of calmodulin (CaM), which is involved in the cytokine-release process. (Schematically based on Figure 4 of Jänig and Baron, 2003; [23] reproduced with permission from *Pain Practice, *2008 [19]).

A still further possible role of phenoxybenzamine in suppression of macrophage involvement relates to the fact that the release of cytokines is mediated by calmodulin [32], and phenoxybenzamine has been shown to have high potency for the inhibition of calmodulin [30, 33, 34]. The possible sites of action of phenoxybenzamine discussed above are summarized in Figure 2.

A recent publication by Chang et al. [35] has added considerable support to the relatively small body of clinical observations that show a possible therapeutic value of phenoxybenzamine for the treatment of CRPS. Their studies identified patterns of genes that were differentially expressed in the Complete Freund's Adjuvant (CFA) animal model. This model results in tactile allodynia and thermal hyperalgesia. Gene map arrays were obtained from RNA extracts of the L4 and L5 dorsal root ganglia of rats that had received intraplantar CFA for 4 days and compared with untreated matched controls. More than 100 genes from the CFA-treated animals were significantly (>1.5-fold; *P* < 0.05) up- or downregulated.

The genes that showed significant changes included many that are involved in immune function, inflammatory response, and neuron growth and survival. The total pattern of gene changes was considered to be a gene “signature” of the CFA model [35]. A search of the Broad Build02 database (and associated matching tool) [36] for pharmacologically active compounds that had strong inverse gene signatures in relation to that of the CFA pathology yielded phenoxybenzamine as one of five compounds with the strongest inverse matches of gene expression.

These investigators next compared phenoxybenzamine with naproxen for its effects on mechanical allodynia in the CFA 4-day model [35]. The drugs and vehicle control were administered by oral gavage on day 4, and behavioral responses to stimuli were measured two hours later. Phenoxybenzamine was found to have approximately equal potency to naproxen in this pain model. With both drugs at a single dose of 10 mg/kg, naproxen reversed the pain sensitivity by 93%, and phenoxybenzamine caused an 82% reversal.

## 5. Side Effects

We have commented previously [19] on the fact that the most common side effects of phenoxybenzamine are related to its *α*-adrenergic antagonist effects. These include postural hypotension, associated reflex tachycardia, and nasal congestion. These effects are relatively minor and are all dose related, and tolerance to these effects, when they appeared, took place after several doses in our own studies [19]. Inhibition of ejaculation has been reported in males, but it would seem that this occurs at higher doses of the drug than the doses used in our experience.

## 6. Controversies in Relation to Carcinogenic Risks 

The labeling for the proprietary oral dosage form of phenoxybenzamine, Dibenzyline, carries a warning regarding possible carcinogenic and mutagenic risks, as observed in *in vitro* and small animal testing (WellSpring Pharmaceutical Corporation, Sarasota, FL). These findings are always of concern and warrant consideration of a risk to benefit evaluation. However, there is little or no evidence that the drug is carcinogenic in humans. Te [37] and Inchiosa and Kizelshteyn [19] have commented on the precedents of widely used drugs (e.g., spironolactone and several of the statin class drugs) that have evidence in animals of carcinogenicity at multiples of human doses. Te [37] has discussed the fact that these concerns have probably limited the valuable application of phenoxybenzamine for the treatment of urinary tract disorders and other pathologies. In his 2002 paper, he estimated that more than 20,000 patients would have been exposed to phenoxybenzamine since it was introduced in 1953. In a review of surveillance data up to 1961, and continuing with adverse event reporting, there were no reports of human cancer that would have appeared to have been related to treatment with phenoxybenzamine [37].

Early in 2013, through the United States Freedom of Information policies, I requested the Adverse Events Reporting data from the FDA for phenoxybenzamine for the past 10-year period. This would largely cover the period since Dr. Te's researches. There were 3 cases in which a malignant disease was recorded in a patient that had received phenoxybenzamine. One case of breast cancer can probably be discounted in regard to an association with phenoxybenzamine since cancer was already suspected at the time that the drug was started. The two other cases involved the use of the drug for urinary obstructive indications. The therapy seemed to have been beneficial for those conditions. Although the adverse event reports from the FDA do not list identifying information, it would appear that they are the same two cases cited in the package insert for Dibenzyline [38, 39]. Both patients had complicated medical histories; it may not be possible to assign cause and effect. It can be noted again that the drug has been in continuous clinical use for 60 years, and there is a paucity of documented evidence of an association with human cancer.

Of particular value in evaluating the question of carcinogenic concerns with phenoxybenzamine is a recent study from the Kaiser Permanente Medical Care Program [40]. This organization is a large chronic care institution with automated records of drugs dispensed and a cancer registry. They analyzed their databases for evidence of carcinogenicity in association with treatment with 9 drugs that are classified by the International Agency for Research on Cancer as probable or possible human carcinogens based on animal or limited human data. Phenoxybenzamine was one of the drugs investigated. It was used in the treatment of a total of 592 patients; there was no statistically significant association with any human cancer.

## 7. Conclusions

Phenoxybenzamine may be a reasonable drug for consideration in the treatment of CRPS, particularly, when a patient has not shown a satisfactory therapeutic response to more conventional therapies. It might also be considered before more invasive therapies, such as intraspinal opioid infusion or spinal cord stimulation, are undertaken. Also, it might be expected that a trial of treatment with phenoxybenzamine for a period of several days may be adequate to detect some improvement in symptoms if the drug is going to have a value for a particular patient [19]. Depending on a patient's current therapies, it may be possible to simply add the drug to the regimen, thereby eliciting some potential additive or synergistic effect. The hypotheses presented here that include potential immunomodulatory/anti-inflammatory effects of phenoxybenzamine appear to be consistent with evidence of an autoimmune contribution to the syndrome [41].

## Figures and Tables

**Table 1:** Summary of clinical trials of phenoxybenzamine for treatment of CRPS.

Predominant syndrome	Total number of patients	Target daily dose range (mg)	Number with favorable clinical outcome	Reference
CRPS II; causalgia	15	40–160	11	Moser et al., 1953 [13]
CRPS II; causalgia	40	40–120	40	Ghostine et al., 1984 [14]
CRPS I	1	20	1	Lefkoe and Cardenas, 1996 [16]
CRPS I and II	33	120	17	Muizelaar et al., 1997 [15]
CRPS I	5	5	5	Malik et al., 1998 [17]
CRPS I	4	3–10	3	Inchiosa and Kizelshteyn, 2008 [19]
